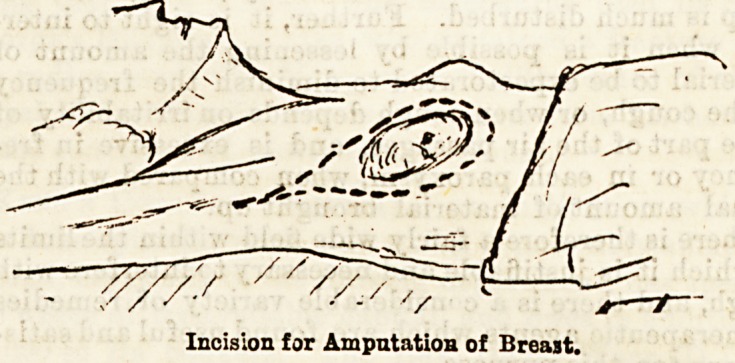# Treatment of Cancer of the Breast

**Published:** 1893-07-22

**Authors:** 


### LONDON HOSPITAL.

### Treatment of Cancer of the Breast.

The treatment of cancer of the breast by the modern surgeon may be summed up shortly by the dictum—*operate early, and operate thoroughly*. In no form of disease or growth is thoroughness more an essential than in the operative treatment of malignant disease.

Coming on so insidiously, and often presenting in its earliest and, from the point of view of treatment, most hopeful stage, some difficulty in diagnosis, the treatment in practice has necessarily to differ considerably from the ideal of theory.

Cancer of the breast being simulated closely by some chronic diseases and non-malignant tumours of the gland, the practical rule has been arrived at to examine at once by incision every doubtful tumour rather than to wait for the course of events to decide the diagnosis. "Hoping for the best" is in tumours of this organ too often a prelude to "preparing for the worst."

No possible harm can arise in these days of antiseptic surgery from removing a tumour, though it may not prove to be of a malignant nature, but nothing can be more unsatisfactory than for a patient to come with well-marked cancer, who a few months before had decided to wait till the nature of the tumour had showed itself; when it does show itself the most favourable time for operating has passed.

The modern operation for cancer of the breast as practised here differs in several material points from the operation as performed even ten years ago.

Clinical experience and microscopic examination have shown that though no gland may be felt enlarged in the axilla, yet in nearly every case of cancer of the breast the axillary glands are affected very early, therefore in every case, no matter how small the tumonr in the breast, the axilla must be cleared out.

When the growth has penetrated at all deeply into the breast tissue, the fascia covering the pectoral muscle, its lower edge and even a superficial layer of the muscle itself is often removed, for it has been found microscopically that in these cases the fascia and most superficial fibres of the muscle are infiltrated with cancer cells, and therefore liable to give rise to recurrence. Along the lower border of the muscle run the lymphatics from the breast to the axilla, which are early impregnated with the cancer cells from the diseased breast.

Very little preparation of the patient for the operation is necessary. The axilla is shaved, the skin cleaned first with soap and water, then ether, finally with 1-40 carbolic lotion, and a compress soaked in the same applied for twelve hours previous to operation.

An aperient is given the night before, and the bowels emptied by an enema the morning of operation. No solid food is given for at least six hours previous to the operation, but a cup of beef tea is allowed a couple of hours before sending the patient to the operation theatre.

When on the table the body below the breasts is well wrapped up in blankets, over which a mackintosh is tucked in round the body, and towels wrung out of warm carbolic lotion are by some placed on the skin and part of the chest that will not be involved in the operation.

The arm on the affected side is extended over the head by an assistant, so as to expose the whole of the axilla.

An incision is made into the tumour to settle the point that it is a cancer before proceeding to remove the breast. This point having been decided, an incision is made, starting from near the sternum, round the lower fold of the breast, up towards the axilla. The breast is drawn down, and another incision is made round the upper fold of the breast to join the first, and prolonged up the axilla to near the insertion of the large pectoral muscle. As the flaps are separated the bleeding is controlled by pressure with the pads of gamgee wrung out of carbolic lotion that are used

Incision for Amputation of Breast.

here instead of sponges. The breast is then separated from the fascia beneath, and, if necessary, the fascia, edge of muscle, and superficial layer of the muscle fibres are removed. Bleeding points that have not stopped are secured in pressure forceps, and attention is turned to the axilla. All the fat in the axilla is removed, with it many small glands that cannot be felt, and of course any larger glands that can be detected; the fingers being used in preference to any other instrument for their removal, care being taken not to exert any undue force when the glands or tissues are in close connection with the axillary vein.

Very few bleeding points require ligature; most of the vessels that have already been secured in pressure forceps will have stopped bleeding by this. A few large ones may require ligature with catgut, or torsion.

The wound is now thoroughly flushed out with warm perchloride of mercury lotion (1-2,000) or carbolic (1-40). If the patient is fat, the lower flap may seem to bag somewhat, when an incision is made through it and a drainage tube passed through so as to drain the most dependent part of the wound, or a piece of drainage tube put into the middle of the primary incision. In the majority of cases drainage tubes have been found unnecessary. The greatest care having been exercised in stopping all bleeding and in removing all clots, the arm is brought down a little, the wound after being well washed out, sutured with silkworm gut, and a few silk sutures, if there is much tension on the flaps when brought together, the wound being pressed as dry as possible before the last sutures are tightened, and pressure being now applied to the flaps by means of pads or sponges.

The skin round the wound having been again cleansed the arm is brought down to the side and across the chest. The wound is dressed either with clean sponges wrung out of carbolic lotion and powdered with iodoform, a very excellent and elastic dressing, or some antiseptic gauze, large pads of cotton wool being used in either case to obtain elastic pressure, and the arm is bandaged very firmly across the chest. The dressings do not need changing till the tenth day, when the greater number of, if not all, the stitches can be removed. The wound will be found united through the whole or greater part of its extent. It is redressed with either antiseptic gauze or a strip of some simple ointment, such as boracic, and bandaged round the chest and shoulder only, it not being necessary at this stage to include the arm in the bandages.

Occasionally, when the extent of skin removed has been such as to prevent the edges from coming easily together, a portion of the wound has to be left to heal up by granulation, being dressed at first with antiseptic gauze and iodoform, later with any simple unirritating or slightly stimulating dressing, such as boracic ointment or very weak nitrate of silver solution.

Certain factors are taken into consideration before deciding whether any case is a fit one for operation. The chief of these are the extent to which the skin is implicated, those cases where there is a cancerous infiltration of the skin spreading over the chest being considered unfit for operation.

The presence of cancer in other organs, or extensive infection and enlargement of the glands above the clavicle, as well as very quickly growing cancer, especially in very fat women, render the performance of an operation almost useless.

Considerable importance has to be attached to the general health, much lung or heart affection, diabetes, or evidence of kidney disease, militate so much against the satisfactory termination of any operation that they become questions of the gravest import when inquiring as to the utility of an operation in a doubtful case, and weighing heavily in the prognosis under any circumstances.

One point of importance remains to be mentioned, the means of alleviating the inevitable suffering when the disease is too advanced to admit of operative interference. Sooner or later opium or morphia in some form has to be given to relieve the pain. Conium is often prescribed for this purpose, but is far inferior to morphia, a small dose of atropine being given with the morphia, hypodermically or by mouth, the atropine appearing to lessen or prevent the sickness and nausea occasionally noticed after hypodermics of morphia alone. Locally no applications lessen the pain to any marked extent. Liniments of belladonna and aconite are the most efficient, but cannot, of course, be used where the skin is ulcerated. The fœtor from the ulcerated surface is a source of great annoyance to the patient and those around her. Frequent washing with Condy's fluid or very weak lotion of chlorinated soda and dressing with an ointment containing iodoform and oil of eucalyptus lessen the odour to a considerable extent. Carbolic lotion, in those cases where its smarting and irritating properties are not too greatly felt, forms an excellent application, the slight numbing effect of the carbolic in lessening the pain, as well as diminishing the odour, being very acceptable to the patient, especially if used in conjunction with the ointment of iodoform and eucalyptus oil.

## Figures and Tables

**Figure f1:**